# Randomized, double-blinded, controlled non-inferiority trials evaluating the immunogenicity and safety of fractional doses of Yellow Fever vaccines in Kenya and Uganda

**DOI:** 10.12688/wellcomeopenres.15579.1

**Published:** 2019-11-20

**Authors:** Derick Kimathi, Aitana Juan, Philip Bejon, Rebecca F. Grais, George M. Warimwe, YEFE and NIFTY vaccine trials teams

**Affiliations:** 1 KEMRI-Wellcome Trust Research Programme, Kilifi, Kenya; 2 Centre for Tropical Medicine & Global Health, Nuffield Department of Medicine, University of Oxford, Oxford, UK; 3 Epicentre, Paris, France

**Keywords:** Yellow Fever vaccine, fractional doses, sub-Saharan Africa, clinical trial

## Abstract

**Introduction: **Yellow fever is endemic in specific regions of sub-Saharan Africa and the Americas, with recent epidemics occurring on both continents. The yellow fever vaccine is effective, affordable and safe, providing life-long immunity following a single dose vaccination. However, the vaccine production process is slow and cannot be readily scaled up during epidemics. This has led the World Health Organization (WHO) to recommend the use of fractional doses as a dose-sparing strategy during epidemics, but there are no randomized controlled trials of fractional yellow fever vaccine doses in Africa.

**Methods and analysis: **We will recruit healthy adult volunteers, adults living with HIV, and children to a series of randomized controlled trials aiming to determine the immunogenicity and safety of fractional vaccine doses in comparison to the standard vaccine dose. The trials will be conducted across two sites; Kilifi, Kenya and Mbarara, Uganda. Recruited participants will be randomized to receive fractional or standard doses of yellow fever vaccine. Scheduled visits will include blood collection for serum and peripheral blood mononuclear cells (PBMCs) before vaccination and on various days – up to 2 years – post-vaccination. The primary outcome is the rate of seroconversion as measured by the plaque reduction neutralization test (PRNT _50_) at 28 days post-vaccination. Secondary outcomes include antibody titre changes, longevity of the immune response, safety assessment using clinical data, the nature and magnitude of the cellular immune response and post-vaccination control of viremia by vaccine dose.

**Ethics and dissemination: **The clinical trial protocols have received approval from the relevant institutional ethics and regulatory review committees in Kenya and Uganda, and the WHO Ethics Review Committee. The research findings will be disseminated through open-access publications and presented at relevant conferences and workshops.

**Registration: **ClinicalTrials.gov NCT02991495 (registered on 13 December 2016) and NCT04059471 (registered on 15 August 2019).

**KEY QUESTIONS**

**What is already known?**

- Yellow fever vaccines should contain a minimum of 1,000 international units (IU) of viral particles per dose
- The use of 587 IU/dose of the Bio-Manguinhos/Fiocruz vaccine administered sub-cutaneously to adults in Brazil was non-inferior to a standard dose of 27,476 IU/dose and protection was sustained for at least eight years.
- WHO recommends the use of fractional dose yellow fever vaccination as an off-label use in response to emergency situations where current vaccine supply is insufficient. The dose fractioning (e.g. 1/5th) should be decided considering the potency of the vaccine batch, the stock available and the suitability of injection devices. The minimal vaccine dose should have a potency of 3,000 IU/dose, but not less than 1,000 IU/dose ^1, 2^.

**What are the key questions the trials are answering?**

- Can all four WHO-prequalified yellow fever vaccines be used as fractional doses with non-inferior immunogenicity when compared to standard vaccine dose?
- Are fractional vaccine doses associated with increased incidence of severe adverse events when compared to the standard vaccine dose?
- What is the minimum yellow fever vaccine dose that is non-inferior to the standard dose?
- Can reduced doses of yellow fever vaccine provide sufficient immunogenicity in infants and children under the age of 5 years?
- Are reduced doses of yellow fever vaccine sufficiently immunogenic in human immunodeficiency virus (HIV)-infected individuals with CD4 counts ≥ 200 cells/ml?
- What is the longevity of the immunity provided by the reduced doses of yellow fever vaccine?

## Introduction

Yellow fever (YF) is caused by a mosquito-borne flavivirus that is endemic in sub-Saharan Africa and tropical South America ^3, 4^. The disease is characterized by a wide range of clinical manifestations, including subclinical, self-limiting or life-threatening illness. Severe disease is characterized by fever, jaundice, haemorrhagic diathesis and multiple organ failure, often ending in death ^5, 6^.

It is estimated that YF causes 200,000 symptomatic cases and 30,000 deaths globally every year ^7^. However, the incidence of YF is believed to be much higher due to underreporting of asymptomatic or mild disease cases that are not identified during epidemics ^8^. More recent estimates for Africa have been in the range of 1.3 million YF infections, of which 180,000 were severe infections resulting in 78,000 deaths ^9^. While there is no specific antiviral treatment for YF, a highly effective and safe vaccine that provides lifelong protective immunity against all seven known genotypes of wild-type YF virus is available ^10– 12^. Reactions to YF vaccine are generally mild and include headache, myalgia, malaise and asthenia in around 10–30% of vaccinees during the first few days after vaccination. Serious reactions are rare and include hypersensitivity reactions to components of the vaccine, YF vaccine-associated neurologic disease and viscerotropic disease ^10, 13, 14^.

The highly effective vaccine is available for routine use in adults and children older than 9 months ^15^. However, during epidemics the vaccine is often used in children from 6 months of age and in pregnant or lactating women ^16^. The YF vaccine is a freeze-dried preparation of the live attenuated YF virus strain 17D that was developed in 1937 and now produced by four WHO-prequalified vaccine manufacturers using sub-strains of 17D (see Table 1) ^17, 18^. The seed virus, derived from the 17D strain, is inoculated into specific-pathogen-free chicken embryonated eggs and after 3–4 days the embryos are aseptically harvested, homogenized and centrifuged to produce bulk vaccine before stabilizing the product. This process is laborious and current capacity to produce increased stock in response to epidemics is limited ^7, 15, 19– 21^.

**Table 1.** Yellow Fever vaccines prequalified by the World Health Organization (August 2016).

Manufacturer	Product Name	Sub-strain	Number of doses per vial
Sanofi Pasteur, France	STAMARIL	17D–204	10
Bio Manguinhos, Brazil	Yellow Fever	17DD	5, 10 or 50
Institut Pasteur de Dakar, Senegal	Stabilized Yellow Fever Vaccine	17D-204	5, 10 or 20
Institute of Poliomyelitis and Viral Encephalitidis, Russian Federation	-	17D-203	2, 5 or 10

WHO recommends that the final vaccine vial/ampoule should contain a minimum of 1000 IU/dose. However, there is no recommendation of a maximum specification and the final dose usually exceeds the minimum specification substantially to account for potential potency losses during manufacture and the three years shelf-life ^15, 22^. The minimum potency recommendation was established in the 1930s and 1940s based on experience with lots that varied in titre and non-standardized measurements. The first international standard between different laboratories was introduced in 2003, where potency results are expressed in IU per dose ^23^. Despite the apparent high vaccine effectiveness observed since thresholds were determined in the 1940s, there is uncertainty regarding the precision with which the minimum dose requirements are known.

A review conducted by PATH identified a number of factors that limit the production of YF vaccines and the relatively long-time period required for vaccine production ^24^. The first is related to the small number of YF vaccine manufactures, owing, in part, to the absence of a stable demand. Competition for production capacity with other vaccines that are economically more attractive is also an issue. Other limiting factors are related to the production process and include the limited number of specific-pathogen-free egg suppliers, gradual depletion of existing seed stocks and a lyophilisation process that can take several days per cycle. These factors together with limited epidemiological surveillance and incomplete national-level reporting, make vaccine need forecasting very difficult ^24^.

With the aim of ensuring an appropriate and coordinated allocation of limited vaccine stocks during epidemics, the International Coordinating Group (ICG) on vaccine provision for YF reserves a stockpile for epidemic response. A stockpile of 2 million doses was established in 2000 and was increased to 6 million in 2014. However, in June 2016, and in response to a large epidemic occurring in Angola and the Democratic Republic of Congo (DRC), 18 million people were vaccinated depleting the stockpile twice ^25^. In July 2016, the ongoing epidemic, together with other emergency demands and the risk of further spread throughout the continent and to Asia, led WHO to develop recommendations for the use of fractional doses of YF vaccine as a dose-sparing strategy ^1^. The recommendation allowed for use of a fifth of a standard dose of the Bio-Manguinhos vaccine. The fractional doses were used in a pre-emptive campaign to vaccinate 7.6 million persons living in the city of Kinshasa. An immunogenicity study was performed as part of that vaccination campaign, but the lack of a control group in that study precludes firm conclusions regarding vaccine efficacy ^26^. Fractional doses were also used to control an epidemic in Brazil owing to vaccine dose shortages ^27, 28^.

The WHO recommendation on the use of fractional doses was based on a limited number of clinical studies and important data gaps remain ^2, 29– 32^. Key research priorities formulated by the WHO include: 1) determining the applicability of fractional dosing to all four WHO-prequalified vaccines, 2) the persistence of vaccine-induced neutralizing antibodies, 3) the performance of fractional doses in young children and in populations in Africa, including HIV-infected individuals and, 4) the incidences of adverse events and serious advance events ^33, 34^. The study protocol described here is aimed at addressing these knowledge gaps through two multicentre randomized, double-blinded, controlled non-inferiority trials in adults and children.

Using vaccines from each of the four WHO-prequalified manufacturers, the first trial (termed YEFE) aims to compare the immunogenicity and safety of a fifth of the dose to the respective standard vaccine dose. The vaccine potencies used in the YEFE trial are as close to the minimum release specifications as possible. Data from the YEFE trial will inform WHO recommendations on the use of a fifth of standard dose of vaccine for immunisation. The second trial (termed NIFTY) aims to compare the immunogenicity and safety of three low vaccine doses (1000, 500 and 250IU/dose) to the standard vaccine dose (>1000IU/dose) using vaccine produced by Institut Pasteur de Dakar in Senegal. The data generated in this study will provide information regarding the definition of the minimal dose and potency requirements of the vaccine with more precision. The study will also provide further confidence in the use of fractional doses of YF vaccine during epidemics.

## Objectives

### Primary objectives

***YEFE***. To determine, for each WHO-prequalified vaccine, whether a fifth of the vaccine dose is non-inferior to the standard dose for each WHO-prequalified vaccine as measured by seroconversion using the PRNT _50_ assay at 28 days post-vaccination in an unvaccinated adult population. 

***NIFTY***. To determine the lowest dose (1000, 500 or 250 IU/dose) of YF vaccine manufactured by Institut Pasteur that is non-inferior to the full standard dose as measured by seroconversion using the PRNT _50_ assay at 28 days post-vaccination in an unvaccinated adult population.

### Secondary objectives

The two trials have some similar secondary objectives as follows:

- To describe the geometric mean PRNT _50_ titre (GMT) at 10 days, 28 days and 1-year post-vaccination of the different doses of the YF vaccine.
- To describe the change in PRNT _50_ titre (i.e. the geometric mean fold increase (GMFI) as a continuous variable) between baseline and day 28 after vaccination with the different doses of the YF vaccine.
- To assess the occurrence of adverse events (AE) over 28 days after vaccination and serious adverse events (SAE) throughout the duration of the studies.

Following results of the main outcome of the studies in adults and data and safety monitoring board (DSMB) recommendations, both studies will assess the immunogenicity and safety of a fractional dose from one vaccine manufacturer (YEFE) and one lower dose (NIFTY) compared to standard dose in children (YEFE and NIFTY) and HIV-infected individuals (YEFE).

The NIFTY trial has additional secondary objectives as follows:

- To assess post-vaccination control of viremia by vaccine dose on samples collected at baseline, and on days 2, 3, 4, 5, 6, 7 and 10 after vaccination.
- To determine the change in T and B cell immune responses between baseline and days 10 and 28 post-vaccination.
- To measure neutralising antibody to other flaviviruses (including dengue, West Nile and zika viruses) on the baseline sample and determine the impact of these antibodies on YF vaccine immunogenicity.
- To determine the change in serum cytokine and chemokine levels between baseline and days 2, 3, 4, 5, 6, 7, 10 and 28 post-vaccination.
- To determine the change in serum cytokine and chemokine levels between baseline and days 2, 3, 4, 5, 6, 7, 10 and 28 post-vaccination.

## Sample size

The studies have been powered to assess non-inferiority of the fractional dose (1/5 ^th^) compared to full standard dose for each manufacturer independently for the YEFE study and of each lower dose of vaccine (1000, 500 and 250 IU/dose) compared to the full standard vaccine dose for the NIFTY study. Sample size calculations were done using PASS and art2bin on Stata version 15.

**Figure 1.**
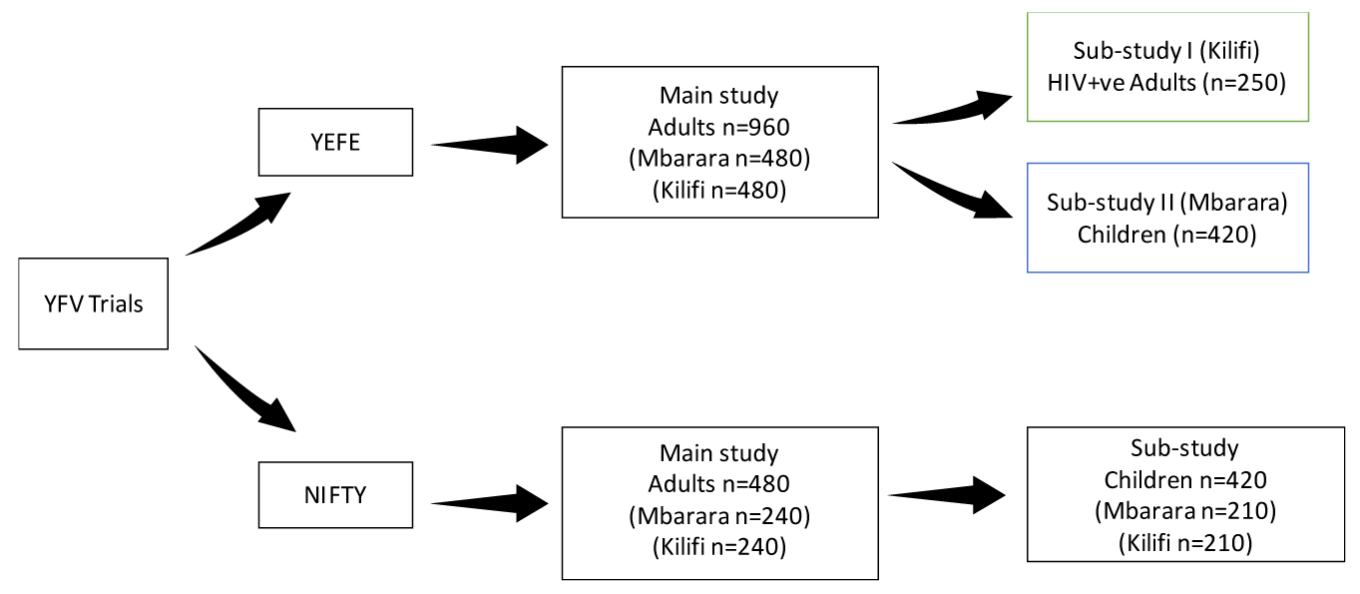
The Yellow Fever vaccine (YFV) trials population.

For the adult studies (YEFE and NIFTY), we assumed a 95% seroconversion rate, 90% power, 2.5% alpha for a one-sided test and a non-inferiority margin of 10%, which gave a sample size of 100 per arm. The 10% non-inferiority margin was chosen in consideration of the public health consequence of a loss of protection but a potential increase in vaccine dosages in a situation where vaccine stocks are insufficient to respond to an epidemic. The sample size was increased by 20% to account for losses to follow up and unevaluable participants with a positive serological response for YF virus at baseline. For the YEFE trial, there are four pairwise comparisons being made for non-inferiority (i.e. full standard dose vs 1/5 ^th^ dose for each vaccine manufacturer). Hence the overall sample size was 960, requiring 480 participants in Kenya and 480 in Uganda. For the NIFTY study a total sample size of 480 will be required for the four vaccine dose groups (i.e. full standard dose, 1000 IU, 500 IU and 250 IU; Figure 1).

For the studies in children (YEFE and NIFTY), we assumed a 90% seroconversion rate (accounting for lower vaccine immunogenicity reported in children ^35, 36^), 90% power, 2.5% alpha for a one-sided test and a non-inferiority margin of 10%, which gave a sample size of 190 per arm. This was increased by 10%; to account for 5% losses to follow up and 5% unevaluable participants with a positive serological response for YF virus at baseline. This gave a total sample size of 420 i.e. 210 in the full standard dose groups and 210 in the fractional or lower dose groups ( Figure 1).

**Figure 2.**
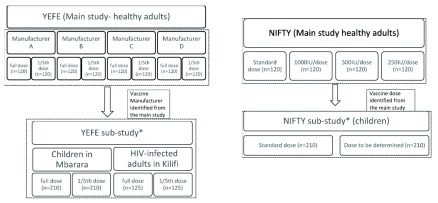
randomization and allocation of the intervention arms. *The sub-studies will be conducted after review of the data for the main outcome by the data and safety monitoring board with decision on one manufacturer or one of the lower doses.

In the YEFE study, assumptions made for the HIV-positive adult sub-study were 83% prevalence of seroconversion, 90% power, 2.5% significance (one-sided test) and a delta of -0.17 (i.e. 17%) ^37^. The 17% non-inferiority margin was determined based on the HIV-positive population being a minority within a larger population and therefore having a smaller overall impact on herd immunity in the population if there is any reduction in immunogenicity, and pragmatism based on the numbers of participants that could likely be recruited. We increased the sample size by 20% to account for loss to follow-up and baseline seropositivity, giving an overall sample size of 250 for the sub-study in HIV-positive adults (see Figure 1 and Figure 2).

## Participants and community engagement

Participating sites are the Kenya Medical Research Institute (KEMRI)-Wellcome Trust Research Programme clinical trials facility in Kilifi, Kenya and Epicentre’s Uganda Research Centre in Mbarara, Uganda. These sites have extensive experience in conducting clinical trials with elaborate community and public engagement strategies ^38^. The studies aim to recruit healthy volunteers, and as such will use locally adapted strategies to inform communities about the study and we will involve district officials or local sub-national health management teams, local administration and community members. A community engagement plan specific for the study was developed at each site. Potential participants will be sensitized for the trials, willing volunteers will be consented before any study specific procedures are undertaken. They will then be screened, enrolled and vaccinated. In Kilifi participants will be recruited in the local community in North Kilifi, while in Mbarara participants will be recruited from communities in Mbarara district.

## Eligibility

The main studies will recruit adults living in Kilifi and Mbarara. The sub-studies will recruit adults living with HIV in Kilifi and children in Mbarara for the YEFE trial, and children in both sites for the NIFTY trial. 

### Inclusion criteria

•      Individuals aged ≥18 to <60 years of age.

◦ For the sub-study, children aged 9 months < 5 years.

•      HIV status

◦ HIV-negative on serological screening OR
◦ HIV-positive adults and children aged > 18 months on serological testing, and no symptoms suggestive of current clinical immunosuppression and CD4 count>200/ml (for adults) and CD4% > 25% (for children aged <5 years) within the last 6 months.

•      Ability to provide informed consent to participate in the study

*Exclusion criteria*

•      Known contraindications to YF vaccination such as allergies to egg protein and chicken products or any component of the vaccine, immunodeficiency due to symptomatic HIV/AIDS or other causes, known thymus disorder, such as thymoma and myasthenia gravis and acute febrile diseases

•      Previous YF vaccination

•      Previous YF infection as determined from history

•      Pregnancy (as determined by a urine test on the proposed day of vaccination) and lactating women

•      Planning to migrate out of the study areas before the end of the study follow-up

•      Planning to travel to a country requiring YF vaccination certificate within the duration of the study

•      Any condition or criteria, including acute or chronic clinically significant abnormality that in the opinion of the investigator might compromise the wellbeing of the volunteer or interfere with the outcome of the study. 

## Informed consent and screening

Following community engagement, the volunteers willing to participate in the studies will be invited to the trial clinic. Before any study-specific procedures are undertaken, the potential participants will provide informed consent. For children (9 months < 5 years of age), consent to participate in the study will be requested from parents or guardians. At least one parent, or guardian, will provide written informed consent for her/his child to participate in the study. A copy of the consent form for the NIFTY trial is available as *Extended data* ^39^. Study documents including the protocol, the informed consent forms will be adapted to the two sites for the conduct of the trials.

All screening procedures will be similar for all participants, regardless of HIV status. On the screening visit, participants will be assessed by a clinician to check the inclusion and exclusion criteria, and a rapid HIV test done according to national guidelines. Relevant demographic information and baseline characteristics will also be collected. HIV-positive volunteers will undergo a CD4 count test during the screening. Screening will also involve assessment for acute febrile disease and other vaccine contraindications, and a urine pregnancy test for female volunteers.

## Randomization, intervention and blinding

Eligible participants will be enrolled and randomized to an arm depending on the study they are recruited to (see Figure 2). They will receive one vaccine. This allocation will be blinded to the participants, the investigators and the study teams, and only the vaccinating nurse and pharmacist will be unblinded. Unblinding will be done at the end of the trial. Unblinding will not be necessary unless, as per DSMB request, for SAE review. The vaccine reconstitution will be done as per the manufacturers’ instructions.

The YEFE adult participants (n=960) will be randomized to receive one of the four vaccines (see Table 1) and one of the two doses (full standard dose or a 1/5 ^th^ of the full standard dose). The allocation ratio used will be 1:1:1:1:1:1:1:1 to one of the eight treatment arms per a computer-generated randomisation schedule. Results for the primary outcome and safety will be reviewed by the DSMB and one manufacturer selected for the sub-studies on children (n=420) and HIV-positive adults (n=250). The allocation ratio for the sub-studies will be 1:1 to standard and fractional dose.

For the NIFTY study, the vaccine manufactured by the Institut Pasteur de Dakar will be used. Adult participants (n=480) will be randomized for vaccination with full standard dose or with 1000, 500 or 250 IU (i.e. 4 arms) with a 1:1:1:1 allocation ratio. Results for the safety and primary outcome of the adult study will then be reviewed by the DSMB, and the lowest non-inferior dose in the adult study selected for assessment in children aged 9 months < 5 years (n=420) in comparison to full standard dose (i.e. two arms) with a 1:1 allocation ratio.

Randomization will be done by randomization booklets with concealed scratch booklets, allocated in order of recruitment and opened on the day of vaccination. These will be prepared by an independent partner. Allocations will be concealed until a member of the unblinded study team scratches the randomization booklet to reveal the participants’ randomization arm. The vaccine will be prepared outside of the view of the participants and the volume concealed using a sticker on the syringe.

## Procedures, follow-up and specimen collection

Participants recruited in YEFE will be followed up for 1 year while in NIFTY, adult vaccinees will be followed up for 2 years, and children for 1 year. Blood samples will be collected at different visits. In the YEFE trial, 4 ml of blood will be collected at screening (baseline), and on days 10, 28 and 365 post-vaccination. For the NIFTY trial, 10 ml and 6ml of blood will be collected for adults and children, respectively, at screening (baseline), and on days 10, 28, and 365 post-vaccination, with an additional sampling of adult participants at 2-years post-vaccination. Further, to monitor post-vaccination viraemia, participants will be randomized for an additional blood collection (4 ml) on either day 2, 3, 4, 5, 6 or 7 after vaccination. The study procedures will be similar across both sites and these are summarized in Table 2.

**Table 2.** Study procedures.

Procedure	Screening Day -30 *	Vaccination Day 0	Day 2,3,4,5,6 or 7 **	Day 10 (±1 day)	Day 28 (±3 days)	Day 365 (±14 days)	Day 730 (±28 days)
Informed Consent	X						
HIV testing	X						
If HIV+, CD4 Test	X						
Pregnancy test	X	X			X		
Demography	X	X					
History and Medical exam	X	X	X	X	X	X	X
Randomization		X					
Vaccination		X					
Blood Samples collected		X	X	X	X	X	X
AEs and SAEs *		X	X	X	X	X	X

*Adverse events (AEs) will be assessed actively until day 28 post vaccine while serious AEs (SAEs) will be assessed for the entire trial period. **Participants in NIFTY will be randomized to provide a blood sample either at day 2,3,4,5,6 or 7 day after vaccination by sparse sampling.

Depending on the results at the end of the studies, we may contact participants to enable long-term follow-up of immunogenicity for the different trials. The long-term follow-up will depend on the initial data from this primary study. An amendment to the protocol with details of any planned long-term follow up will be made and submitted to the various regulatory authorities for review and approval.

## Outcomes

### Laboratory assessments

Blood samples will be processed to serum (YEFE and NIFTY) and PBMCs (NIFTY) within 8 hours of collection. Serum isolated will be used for PRNT _50_ assay, considered the most sensitive and specific test for quantification of neutralizing antibodies and is the reference method for assessing immune response after vaccination ^40, 41^. Viral RNA will be isolated from serum for detection YF vaccine virus by qRT-PCR on days 0, 2, 3, 4, 5, 6, 7 and 10 (see Table 2) ^42, 43^. This sparse sampling approach will allow detection and modelling of YF vaccine virus levels in blood, by study arm, whilst minimizing the number of samples taken per individual ^44, 45^. In this study we will also characterise the cellular (T and B cell) immune responses to YF vaccination in both adults and children. Whilst these responses have been previously characterised, there is very little data from populations in Africa, including knowledge gaps on how the cellular immune kinetics change with vaccine dose (full vs. lower doses) and age ^46^. We will therefore isolate PBMCs from the blood samples collected at baseline, day 10 and day 28 post-vaccination and use these for assessment of cellular immune responses by flow cytometry. To complement the cellular immune assessments by flow cytometry, we will measure the chemokine and cytokine response to vaccination in serum samples collected at baseline and on days 2, 3, 4, 5, 6, 7, and 10 using a multiplex immunoassay system.

High seroprevalence of antibodies to flaviviruses (e.g. dengue virus) has been reported in East Africa ^47– 49^, raising the question whether presence of antibodies to other flaviviruses at the time of immunization has an impact of YF vaccine immunogenicity. Baseline samples in the NIFTY study will therefore be evaluated for presence of antibodies to other flaviviruses (including dengue, West Nile and Zika virus) and their association with YF vaccine immunogenicity assessed.

### Safety and adverse events assessment

Active assessment of AEs will be done in all participants up to day 28. Passive assessment will continue during the follow-up period and will be reported. Each AE will follow a causality assessment. For this, the investigator will determine all contributing factors applicable to each event. These contributing factors will be documented and reported. Every effort will be made by the investigator to explain each AE/SAE and assess its causal relationship to administration of the study vaccine.

### Analysis of outcomes

The intention-to-treat (ITT) population will comprise all randomized participants who received a dose of a study vaccine and that have at least one post-vaccination blood sample. The per protocol (PP) population will include randomized participants who have a blood sample at baseline and 28 days (±3 days) post-vaccination, who are seronegative (PRNT _50_ <1:10) to YF at baseline, and for whom the eligibility criteria were correctly applied. The safety population will include all subjects who received a study vaccine.

The primary analysis will be a pairwise statistical comparison of the rate of seroconversion at day 28 between full dose and fractional dose of each vaccine manufacturer for the YEFE trial, and between the standard dose and each lower dose of vaccine for the NIFTY trial using a non-inferiority test with a margin of non-inferiority of 10% in the PP population. Seroconversion will be defined as a ≥4-fold rise in PRNT _50_ titre between day 0 and day 28 samples. Any PRNT _50_ value reported as below the limit of quantification (LOQ) (e.g. <1:10) will be converted to LOQ/2. Thus a 4-fold rise for a subject who is <1:10 at baseline, is a titre of 20. Each immunogenicity assessment will be a pairwise comparison of the full dose and fractional (1/5 ^th^) or full dose and each lower dose within one study population (i.e. YEFE or NIFTY).

Secondary analyses will include assessment of seroconversion in the ITT population as a whole, on the subset of the ITT population with baseline seropositivity to YF, and in the subset of the PP population with no reported history of flavivirus infection. Geometric mean PRNT _50_ titre (GMT) and GMT fold increase (GMFI) and corresponding 95% confidence intervals (CI) on day 0 and 28 will be calculated. A test of non-inferiority will be performed for the difference in GMT and GMFI between the full dose and each lower dose group. Titres will be graphically represented by reverse cumulative distributions obtained by plotting, for each possible value of the titre (abscissa), the proportion of subjects with a titre greater than this value.

Lower vaccine doses may change the kinetics of antibody response. The assessment of seroconversion rates, GMT, and GMFI 10 days after vaccination in the ITT population will provide important information in the context of low dose vaccine usage in outbreak response. These three immunogenicity outcomes will also be assessed at 1 year and 2 years post-vaccination in the ITT population to confirm a lasting effect of full and low dose vaccination.

Relationships between seroconversion and vaccine immunogenicity (PRNT _50_ GMT and GMFI) will be related to frequencies of specific T and B cell subsets measured by flow cytometry, chemokine and cytokine levels in sera and neutralising antibody levels to other flaviviruses. Correlations between vaccine viremia and immunogenicity will be assessed across the different vaccine dose strata. Comparisons of these immune and viremia kinetics will be made between the adult and children trial participants, whilst accounting for the administered vaccine dose. 

Adverse events occurring during the study follow up period will be analysed and compared between groups. This will be a descriptive analysis and will include all AEs up to 28 days post-vaccination, and SAEs that occurred any time during study follow-up.

A copy of the data management plan, alongside the statistical analysis plan, is available as *Extended data* ^39^.

## Data monitoring committees

The DMCs will be appointed by the sponsor and be composed of three independent members with expertise in clinical trials and vaccinology. The trials’ DMCs will be independent and will meet to review the safety data and reports submitted. After the first phase of each study, the DMCs will meet to review the data and make a decision on the fractional dose to be used in the second phase. The DMCs may also have ad hoc meetings convened by the chair and/or the sponsor. The DMCs will be empowered to recommend pausing or stopping the trial to the sponsor, and to request any additional information pertaining to participant safety that is considered necessary. The remit and functions of the DMCs are described in the DMC’s Charter (available as *Extended data* ^39^).

## Status of the trials as of October 2019

At the time of submitting this manuscript, the YEFE main study had completed recruitment (n=960) and one-year follow-up at both sites and laboratory analysis was ongoing. YEFE sub-studies recruiting adults living with HIV in Kilifi (n=250) and children 9 months to <5 years in Mbarara (n=420) had completed recruitment and 28 days follow-up visits. The NIFTY study had just begun in Kilifi, Kenya and Mbarara, Uganda, after having obtained all approvals.

## Ethics and dissemination

The trial protocols have been approved by the relevant ethics and regulatory authorities in Kenya and Uganda. In Kenya, approvals have been received from the KEMRI Scientific and Ethics Review Unit (SERU), and the Kenya Pharmacy and Poisons Board (PPB). In Uganda, approval has been granted by the Mbarara University of Science and Technology’s Ethics Review Committee (MUST-REC), Uganda National Council of Science and Technology, and the National Drug Authority. Further approvals have been received from the Oxford Tropical Research Ethics Committee (OxTREC) at the University of Oxford, and the YEFE trial also received approval from the WHO Ethics Review Committee.

The research findings will be disseminated as open-access journal publications and presented at relevant conferences and workshops. Further, the results will be shared with the participating communities in workshops and engagement forums.

## Data availability

### Underlying data

No underlying data are associated with this article.

### Extended data

Figshare: Randomized, double-blinded, controlled non-inferiority trials evaluating the immunogenicity and safety of fractional doses of Yellow Fever vaccines in Kenya and Uganda. https://doi.org/10.6084/m9.figshare.10283048.v2 ^39^.

This project contains the following extended data:

- YEFE Statistical Analysis Plan v1.1-clean-signed (statistical analysis plan).
- NIFTY Protocol Version 1.3 04032019_final (protocol for NIFTY trial).
- NIFTY Informed_Consent_storage of biological_materials_v1 21 Nov 2018_draft_DK (participant consent form for storage of biological materials).
- NIFTY adult ICF English Version 1.3 04032019 (consent form for NIFTY trial).
- DMP_NIFTY V1.0 (data management plan for NIFTY trial).
- Ethical approval documents

### Reporting guidelines

Figshare: Randomized, double-blinded, controlled non-inferiority trials evaluating the immunogenicity and safety of fractional doses of Yellow Fever vaccines in Kenya and Uganda. https://doi.org/10.6084/m9.figshare.10283048.v2 ^39^
