# Supplementary material for: Randomized, double-blinded, controlled non-inferiority trials evaluating the immunogenicity and safety of fractional doses of Yellow Fever vaccines in Kenya and Uganda
Source: Wellcome Open Res. 2019 Nov 20;4:182. [Version 1] doi: 10.12688/wellcomeopenres.15579.1 (PMC6971842; doi:10.12688/wellcomeopenres.15579.1)
Supplement: Peer review history

# Peer review: Reviewer response for version 1

**DOI:** 10.21956/wellcomeopenres.17060.r37128

**Published:** 2019-12-19

**Authors:** Akira Homma

**Affiliations:** 1 Bio-Manguinhos (Institute of Technology in Immunobiology), Ministry of Health, Oswaldo Cruz Foundation (Fiocruz), Rio de Janeiro, Brazil

General Comment:

 The both trials have been very well described in this manuscript, considering the common objectives and methodology, while describing each one's particularities. These trails have been well designed and their results will have a big potential to greatly contribute to the yellow fever mass vaccination policy.

 Specific comments:

 Regarding the dose comparison: full dose; 1/5 dose; 1.000 IU; 500 IU; 250 IU. There is a very few virological difference among 1.000; 500 and 250 IU. My suggestion is to use: full dose; 1/5 dose; 1.000 IU; 250 IU and 50 IU. With these dosages you will have a chance to get the end-point of dosage.

 Abstract: “Introduction” - The authors mention that yellow fever vaccine provides “life-long immunity following a single dose vaccination”. My suggestion is to mention the results of some cross-sectional studies (conducted by Collaborative Group for Studies of Yellow Fever Vaccine in Brazil) which show a decrease in humoral and cellular immunity a few years after vaccination in children and adults.

 Introduction (page 3 – 3 ^rd^  paragraph): According to the text, the “four WHO-prequalified vaccine manufacturers using sub-strains of 17D”, but the Bio-Manguinhos YFV uses de 17DD sub-strains (the information in Table 1 is correct).

 Eligibility: The sub-studies in children include ages from 9 months to 5 years. Considering the difference in YFV immunogenicity in children vaccinated before 2 years of age, which is demonstrated in several studies, and the WHO recommendation not to use the fractional dosing in children under 2 years of age, we suggest to the authors to justify why choose the 5 year age upper limit and if the stratified analysis is planned to both groups: 9 months - 2 years and >2 years 5 years.

I confirm that I have read this submission and believe that I have an appropriate level of expertise to confirm that it is of an acceptable scientific standard.

## References

[1] : Duration of post-vaccination immunity against yellow fever in adults. *Vaccine*.2014;32(39) : 10.1016/j.vaccine.2014.07.021 4977-84 10.1016/j.vaccine.2014.07.021 25090646

[2] : Booster dose after 10 years is recommended following 17DD-YF primary vaccination. *Hum Vaccin Immunother*.2016;12(2) : 10.1080/21645515.2015.1082693 491-502 10.1080/21645515.2015.1082693 26360663PMC5049740

[3] : Duration of post-vaccination humoral immunity against yellow fever in children. *Vaccine*.2019;37(48) : 10.1016/j.vaccine.2019.09.051 7147-7154 10.1016/j.vaccine.2019.09.051 31590934

[4] : Short-Lived Immunity After 17DD Yellow Fever Single Dose Indicates That Booster Vaccination May Be Required to Guarantee Protective Immunity in Children. *Front Immunol*.2019;10: 10.3389/fimmu.2019.02192 2192 10.3389/fimmu.2019.02192 31616412PMC6775283

# Peer review: Reviewer response for version 1

**DOI:** 10.21956/wellcomeopenres.17060.r37125

**Published:** 2019-12-02

**Authors:** Anna H. E. Roukens

**Affiliations:** 1 Department of Infectious Diseases, Leiden University Medical Centre, Leiden, The Netherlands

General comment:

 The protocol is written very well and the end points and analysis are formulated clearly. The purpose of these two trials is clear and the results will provide a very important contribution to the policy on the use of fractional dose yellow fever vaccine in adults, children and hiv-infected persons.

 In addition, the authors will investigate the cellular immunity, which will give more insight in the understanding of the development (or lack) of immunity against yellow fever.

 Specific comments:

 Abstract

 Methods and analysis: Last sentence: what do the authors mean by the secondary outcome post-vaccination control of viremia by vaccine-dose?

 Do they mean that vaccine-induced viremia will be measured?

 Page 3 table 1: The yellow fever vaccines used are presumably from different batches and thus will contain different amounts of vaccine virus (expressed as IU/dose). The investigators should measure the IU/dose per batch and report this if it contributes to the interpretation of the results.

 Page 4 Objectives

 Primary objectives: Seroconversion using the PRNT50 is measured, does this include the conversion to IU/L, to enhance comparison with other trial outcomes?

 Secondary objectives: In the abstract, the authors state that the follow-up will be two years after vaccination, in the description of the secondary objectives this is one year. Please adjust. (Table 2 indeed has a time point of 2 years post-vaccination).

 The investigators should make an effort to extend the follow up period beyond 2 years, as it has recently been shown that especially in children vaccinated with yellow fever vaccine, a progressive time-dependent decrease in neutralising antibodies was observed as early as 2 years after vaccination, and declined toward critical values (below 60%) at time-spans of > 4 years ^1^.

I confirm that I have read this submission and believe that I have an appropriate level of expertise to confirm that it is of an acceptable scientific standard.

## References

[1] : Short-Lived Immunity After 17DD Yellow Fever Single Dose Indicates That Booster Vaccination May Be Required to Guarantee Protective Immunity in Children. *Front Immunol*.2019;10: 10.3389/fimmu.2019.02192 2192 10.3389/fimmu.2019.02192 31616412PMC6775283
